# EEG responses to standardised noxious stimulation during clinical anaesthesia: a pilot study

**DOI:** 10.1016/j.bjao.2022.100118

**Published:** 2022-12-30

**Authors:** Malte Anders, Björn Anders, Elias Dreismickenbecker, Darren Hight, Matthias Kreuzer, Carmen Walter, Sebastian Zinn

**Affiliations:** 1 Clinical Development and Human Pain Models, Fraunhofer Institute for Translational Medicine and Pharmacology ITMP, Frankfurt, Germany; 2 Center for Pediatric and Adolescent Medicine, Childhood Cancer Center, University Medical Center Mainz, Mainz, Germany; 3 Department of Anaesthesiology and Pain Medicine, Inselspital, Bern University Hospital, University of Bern, Bern, Switzerland; 4 Department of Anesthesiology and Intensive Care, School of Medicine, Technical University of Munich, Munich, Germany; 5 Goethe University Frankfurt, University Hospital, Clinic for Anesthesiology, Intensive Care Medicine and Pain Therapy, Frankfurt am Main, Germany

**Keywords:** EEG, general anaesthesia, nociception, pain, pain-related evoked potentials, pilot study, tetanic stimulation

## Abstract

**Background:**

During clinical anaesthesia, the administration of analgesics mostly relies on empirical knowledge and observation of the patient's reactions to noxious stimuli. Previous studies in healthy volunteers under controlled conditions revealed EEG activity in response to standardised nociceptive stimuli even at high doses of remifentanil and propofol. This pilot study aims to investigate the feasibility of using these standardised nociceptive stimuli in routine clinical practice.

**Methods:**

We studied 17 patients undergoing orthopaedic trauma surgery under general anaesthesia. We evaluated if the EEG could track standardised noxious phase-locked electrical stimulation and tetanic stimulation, a time-locked surrogate for incisional pain, before, during, and after the induction of general anaesthesia. Subsequently, we analysed the effect of tetanic stimulation on the surgical pleth index as a peripheral, vegetative, nociceptive marker.

**Results:**

We found that the phase-locked evoked potentials after noxious electrical stimulation vanished after the administration of propofol, but not at low concentrations of remifentanil. After noxious tetanic stimulation under general anaesthesia, there were no consistent spectral changes in the EEG, but the vegetative response in the surgical pleth index was statistically significant (Hedges' *g* effect size 0.32 [95% confidence interval 0.12–0.77], *P*=0.035).

**Conclusion:**

Our standardised nociceptive stimuli are not optimised for obtaining consistent EEG responses in patients during clinical anaesthesia. To validate and sufficiently reproduce EEG-based standardised stimulation as a marker for nociception in clinical anaesthesia, other pain models or stimulation settings might be required to transfer preclinical studies into clinical practice.

**Clinical trial registration:**

DRKS00017829.

Excessive postoperative pain can be considered a complication of general anaesthesia that can impair quality of life.^1^, ^2^, ^3^, ^4^, ^5^ In order to improve patient outcomes, we should aim for personalised general anaesthesia with optimised analgesia.^6^^,^^7^ Existing electroencephalography (EEG)-based monitoring devices, such as the bispectral index (BIS), generate a dimensionless index scale to reflect the depth of anaesthetic.^8^^,^^9^ The utility of processed EEG (pEEG) data is still a matter of debate. Proprietary algorithms heavily compress the large amount of information encompassed by the frequency, amplitude, and phase of the raw EEG,^10^ such that specific physiological, pathophysiological, and pharmacological signatures in the EEG are lost. Also, drugs may have apparently paradoxical effects on the pEEG (e.g. ketamine can lead to falsely high values or neuromuscular blocking agents without any hypnotic properties can lead to a decrease in the index).^11^ However, specific patterns can be distinguished by observing the raw EEG during anaesthesia, but such analyses are within the capabilities of most anaesthesia providers.^12^ In addition, the usual pEEG indices do not specifically track the nociceptive component (i.e. the brain's reaction to a noxious stimulus).^13^ At a moderate depth of general anaesthesia or during deep sedation, noxious stimulation, such as tracheal intubation or skin incision, may cause an increase in EEG beta power (beta arousal).^14^ Some EEG-based monitoring systems can detect such arousal,^15^^,^^16^ which may also be accompanied by movement of the patient.^17^^,^^18^ At deeper levels of anaesthesia, the EEG can show a different set of changes to a noxious stimulus, which is either a decrease of prevailing alpha oscillations caused by a thalamocortical loop absent of afferent input^19^ that may reflect adequate anaesthesia,^20^ or an increase in amplitude of delta oscillations. These changes are not reliably tracked by pEEG monitors and can even lead to incorrectly low indices.^12^

Non-cortical, biomarkers of vegetative functions, which are used to track nociception, such as blood pressure and heart rate, are sensitive, but not very specific to noxious events. Some objective nociceptive biomarkers such as the surgical pleth index (SPI, GE Healthcare, Helsinki, Finland)^21^^,^^22^ are a haemodynamic surrogate of the autonomic response, monitoring the balance between nociception and antinociception.^23^, ^24^, ^25^

EEG studies on pain in awake, healthy subjects use highly standardised, time-locked, painful stimulations to obtain insights into nociceptive processes. In contrast, EEG studies of nociception during clinical anaesthesia in a heterogeneous patient population often consider only the invasive, intense noxious stimuli inherent to the surgical procedure, such as skin incision: these are more difficult to compare.^15^ Whereas noxious events may alter the EEG in different ways during general anaesthesia^26^ and are still being researched, somatosensory processing as a marker for the perception of noxious stimulation can be tracked using the EEG in healthy, awake participants and is extensively described in the literature.^27^, ^28^, ^29^, ^30^

In this feasibility study, we aimed to determine to what extent (1) conventional evoked responses in the EEG after standardised noxious stimulation can still be identified in patients undergoing general anaesthesia with propofol and remifentanil during clinical anaesthesia and (2) if more intense and prolonged standardised tetanic stimulation alters the cortical function in the EEG or the vegetative reaction in the SPI in a reproducible fashion.

## Methods

### Study protocol and patients

The local ethics committee (‘Ethik-Kommission des Fachbereichs Medizin’) at the Goethe University Hospital Frankfurt approved our study protocol in a written statement under the processing number 6/19. We registered the study with the German Clinical Trials Register under the trial ID DRKS00017829 on 3 February 2020. This study conformed to the standards set by the Declaration of Helsinki. We explained the study protocol to the patients during the standardised anaesthesia informed consent interview. If the patients were willing to take part in the study, we obtained their written consent. The study was carried out at the Goethe University Hospital in Frankfurt, Germany. We enrolled patients who were scheduled for orthopaedic surgery with a low risk of complications (ASA 1 and 2). Our patients were required to be at least 18 yr old, to not suffer from chronic pain, and to have not taken any opioids within 24 h of surgery. We also excluded patients with polyneuropathies, current ongoing drug abuse, neuro-psychological disorders, and pregnant women. Our results were collected during routine clinical anaesthetic management before surgical incision.

### Induction and maintenance of general anaesthesia

**Fig. 1:**
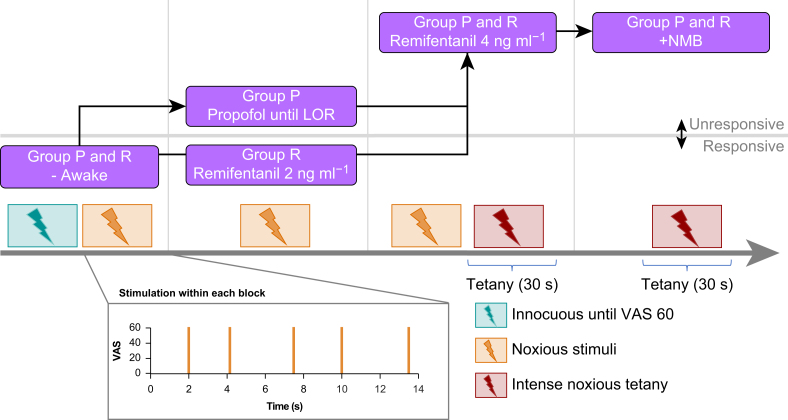
Study flow. Phase-locked stimuli were applied (1) in awake patients, (2) at induction with propofol alone or with remifentanil alone, and (3) at steady state with a combination of propofol and remifentanil. Tetanic stimulation was performed in our patients during stable anaesthesia. LOR, loss of responsiveness; VAS, visual analogue scale; NMB, neuro muscular block.

We induced and maintained total intravenous general anaesthesia using target-controlled infusions of propofol and remifentanil, using a B. Braun Space® pump system (B. Braun SE, Melsungen, Germany). We titrated propofol to achieve loss of responsiveness (LOR—the absence of a visible ocular reaction to a trapezius muscle squeeze). Our target effect-site concentrations for maintenance were 3 μg ml^−1^ for propofol (Schnider model)^31^ and 2–4 ng ml^−1^ for remifentanil (Minto model).^32^^,^^33^ A swift induction and adequate level of anaesthesia as per clinical routine were prioritised in every case, resulting in the necessity for higher initial propofol doses (Table 1). During induction, patients either first received propofol until LOR followed by remifentanil (Group P) or remifentanil at a low dose (2 ng ml^−1^) followed by propofol up to LOR and a subsequent increase in remifentanil concentration up to 4 ng ml^−1^ and rocuronium (0.6 mg kg^−1^) for neuromuscular block (Group R) (Fig. 1). No other CNS-acting drugs were administered during the induction phase.

**Table 1:** Patient characteristics and propofol effect site concentrations. The baseline pain score was evaluated using a 0–100 mm visual analogue scale.

		Group P			Group R	
	Mean	Min	Max	Mean	Min	Max
Age	41	23	67	36	18	66
Sex		5× Male 0× Female			7× Male 4× Female	
Weight (kg)	84	65	97	81	65	105
Height (m)	1.79	1.68	1,88	1.79	1.66	1.98
BMI (kg m^−2^)	26.1	21.2	28.0	25.3	18.4	34.5
Effect site conc. propofol at induction (μg ml^−1^)	6.60	4.99	8.70	3.38	2.90	4.00
Effect site conc. propofol for maintenance (μg ml^−1^)	3.78	3.19	4.70	3.03	2.55	3.37
Baseline pain score (mm)	0	0	0	18	0	80

### EEG/SPI recordings and pre-processing

We used 32 active EEG electrodes (g.Tec g.SCARABEO, Guger Technologies, Schiedlberg, Austria) attached to a g.Tec amplifier (g.HIamp), arranged in the 10–20 system. We chose AFz as the reference and grounding point during the recording phase and changed it to an average reference over all 32 electrodes during off-line pre-processing. For EEG analysis, we utilised MATLAB 2021a (Natick, MA, USA) and EEGLAB v2021.1.^34^ Using EEGLAB for pre-processing, we downsampled the data from 512 to 256 Hz (*pop_resample*), bandpass filtered the data (1–100 Hz, *pop_eegfiltnew*), filtered the line noise (*pop_cleanline*), and performed artifact rejection (*pop_clean_rawdata*). The latter toolbox applies an automated process called artifact subspace reconstruction to filter out artifacts in continuous EEG data; our threshold was set to 20 standard deviations from the cleanest part of the data.^35^, ^36^, ^37^ We extracted the epochs containing the painful stimuli from −1 s to +2 s around the stimulus onset and the epochs containing the tetanic stimulation from −10 s to +40 s around the stimulus onset.

For analysis of the spectrogram, we utilised the MATLAB function *pwelch* with a 5 s window length and a 0.5 s window shift from the signal processing toolbox. For analysis of the common frequency bands, we averaged delta (1–4 Hz), alpha (8–12 Hz), and beta (13–25 Hz) oscillations over the respective frequency range.^38^ For analysis of the event-related potentials (ERPs), we relied on custom-made MATLAB scripts. We also analysed the event-related spectral perturbation (ERSP), a measure that evaluates the relative spectral changes *vs* time against an individual baseline for each patient, as in our previous publication.^62^ For the calculation of the ERSP, we applied the EEGLAB *newtimef* function. For normalisation, we used a divisive baseline from −1 s to 0 s pre-stimulus,^39^ a resolution of 200 frequency points and a resolution of 400 points in time for the phase-locked data. For the tetanic stimulation, we set the baseline to −10 s to 0 s pre-stimulus, the frequency resolution to 1596, and the resolution in time to 6000 time points. The *newtimef* incorporates both a short-term Fourier transform and a wavelet transform; the wavelet transform was applied with three cycles at the lowest frequency (3 Hz for the ERP data and 1 Hz for the tetanic stimulation) and 100 Hz for the highest frequency. In this manuscript, we show the data at the Cz electrode location.^28^ For analyses of the frontal region, we show density spectral array (DSA) for the average of frontal electrode positions Fp1, Fp2, and F9.^20^^,^^40^

The SPI was recorded using an additional GE Carescape B450 monitoring system (GE Healthcare, Solingen, Germany). The data were extracted using the open source software Vital Recorder and stored offline.^41^

For the objective comparison of the respective anaesthetic level, the BIS was subsequently calculated (Medtronic GmbH, Meerbusch, Germany). We replayed the original EEG to a BIS monitor with an NI USB-6343 DAQ card (National Instruments, Austin, TX, USA) which converts the EEG into a continuous signal.^42^ We extracted trend data with 1 s^−1^ from the BIS via a.spa file generated during playback via USB.

### Painful stimuli

All patients received painful stimuli during consciousness before anaesthesia (Fig. 1) using a Digitimer DS7A constant current stimulator (Digitimer Limited, Welwyn Garden City, UK) synchronised to our EEG device via the +5 V TTL output. One electrical shock consisted of four consecutive single electrical stimuli with a pulse width of 200 μs and a maximum voltage of 400 V. The inter-stimulus interval between those four stimuli was 5 ms. Although we administered four concurrent stimuli, because of the short overall duration, they were perceived as one long electrical shock. To determine the required stimulus current, we increased it from 1 mA to a value where the patient rated the subjective pain as being approximately 60/100 on a verbal scale.

We administered a train of five single electrical shocks with a pseudo-randomised inter-stimulus interval of 3–5 s at different stages of anaesthesia induction after reaching stable target concentrations (Fig. 1). These stages were while the patient (1) was awake, (2) had received either propofol at levels required to be non-responsive (Group P) or remifentanil at a target concentration of 2 ng ml^−1^ (Group R), and (3) had received propofol and remifentanil combined, at propofol levels that were required to maintain unconsciousness and a remifentanil target concentration of 4 ng ml^−1^.

### Tetanic stimulation

For tetanic stimulation, we applied 1500 electrical stimuli with a current of 50 mA, an inter-stimulus interval of 20 ms (50 Hz), a maximum voltage of 400 V, a pulse width of 200 μs, and a total duration of 30 s. Tetanic stimulation after LOR was only carried out if the following conditions were met: remifentanil was used at a concentration of 4 ng ml^−1^, the patient belonged to the group that received remifentanil first, and if it did not unduly delay the surgery.

### Statistics

Here we investigate the applicability and transferability of noxious stimulations during routine clinical practice in patients.^43^ In preclinical studies with comparable stimulation patterns, 10 patients were included.^26^ To compensate for uncertainties in effects as a result of the clinical anaesthetic regimen, our exploratory study was planned to include at least 15 patients who received the weakest stimulus awake and 10 patients who received the strongest stimulus, as is done in preclinical studies.^26^^,^^44^

For the intra-subject analysis of the event-related spectral perturbation (ERSP), we calculated effect size using the area under the receiver operating characteristics (AUROC) curve by the MATLAB toolbox MES.^45^ We applied a 1000-fold bootstrap to the 95% confidence intervals (CIs) and only reported results as being significant if the intervals did not include 0.5.^45^ For dichotomous data, this approach is equivalent to the non-parametric Wilcoxon–Mann–Whitney test or the prediction probability (pk).^46^ We compared the changes between the two conditions (awake *vs* fully sedated) with a fixed value of 1 using the *auroc* function of the MES toolbox. An AUROC value of 0.5 indicates a completely random relationship between the conditions, whereas a value of AUROC=0 or AUROC=1 indicates a perfect separation. We further ranked our AUROC values according to a traditional points system with an AUROC value of 1–0.9/0–0.1 being excellent, an AUROC value of 0.9–0.8/0.1–0.2 being good, an AUROC value of 0.8–0.7/0.2–0.3 being fair, an AUROC value of 0.7–0.6/0.3–0.4 being poor, and the remaining AUROC values as being fails.^47^ For comprehensibility, we only extracted the maximum ERSP and AUROC values from our data as they were not dependent on the chosen size of the window. To avoid multiple comparisons over time in the cases of ERSP analysis, we only reported results as being significant if they occurred in a cluster of at least 4×4 pixels in size. For the AUROC values, we have shown the 95% CIs in square brackets.

To calculate the effect size of index values before and after tetanic stimulation, we used paired tests with the Hedges' *g* function of the MES toolbox. To evaluate statistical differences without previous power calculation, we applied a paired non-parametric Wilcoxon signed-rank test and reported results as being significant if their *P*-value was <0.05. For the mean values, we have shown the standard deviations in brackets, whereas for the median values, we have shown the 25% and 75% percentiles in square brackets. To avoid false-positive results as a result of multiple comparisons over time in the ERP analysis, we only reported results as being significant if at least three adjacent time points had a *P-*value <0.05.^48^^,^^49^

## Results

### Patients

Twenty-five eligible patients agreed to take part in the study and gave their written consent. Organisational constraints (e.g. alterations of the surgical timetable at short notice), meant that we were only able to record data from 17 of the 25 patients, six in Group P and 11 in Group R (Table 1). All patients were scheduled for elective surgery because of orthopaedic trauma (open reduction and internal fixation of fractures, removal of metalwork, anterior cruciate ligament reconstruction, meniscus repair, knee arthroscopy) or cartilage disease (knee arthroscopy/synovectomy). Before surgery, patients reported a median pain score of 5 mm at rest on a visual analogue scale (VAS, 0–100 mm) with a wide range [min 0, max 80] (Table 1). Besides the expected decrease in blood pressure and heart rate, no patient in the P or R groups experienced further, clinically relevant haemodynamic impairment after LOR that required the administration of catecholamines.

### Evoked response after phase-locked noxious electrical stimulation

All 17 patients tolerated the painful cutaneous electrical stimulation while awake. The overall median current (obtained in the awake subject before the administration of propofol or remifentanil) associated with a subjective pain rating of 60/100 was 17 mA [11.38; 22.0]; 20.5 mA [10.5; 22.0] in Group P and 16.5 mA [11.63; 21.88] in Group R. The overall median subjective pain rating for consecutive stimulation of five painful bursts was 55 [50; 60]: 50 [40; 60] in Group R and 40 [30; 50] in Group P. In Group R, the subjective pain ratings for the five consecutive electrical bursts decreased to a median value of 50 [42.5; 60.0] after the administration of remifentanil (no propofol) at a target effect-site concentration of 2 ng ml^−1^: the difference compared with pre-remifentanil rating was not statistically significant (*P=*0.094). No other subjective pain ratings could be obtained as the patients were unresponsive during the other conditions.

**Fig. 2:**
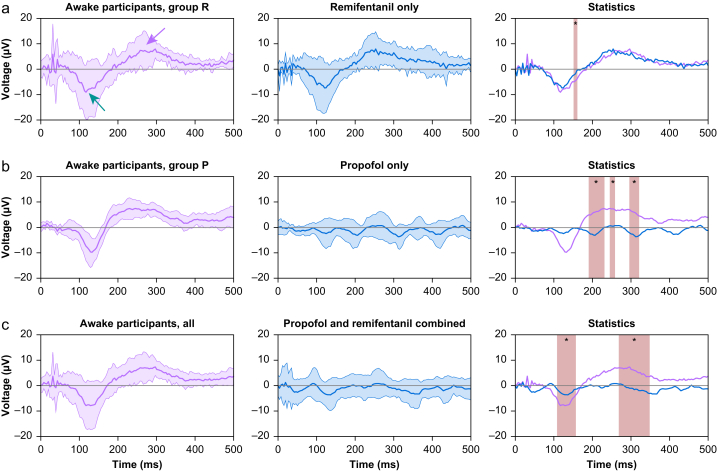
Evoked response after painful noxious electrical stimulation in awake and sedated patients. The top row (a) shows Group R (i.e. the group that started the induction of anaesthesia with remifentanil). The middle row (b) shows Group P (i.e. the group that started the induction of anaesthesia with propofol). The bottom row (c) shows all patients of Groups R and P combined in an awake state and after the administration of both propofol and remifentanil later in time after a steady state of general anaesthesia was achieved. The right-hand side column shows the statistical comparison between the left and middle columns, whereas a red box indicates a statistically significant difference in at least three adjoined points in time. The blue arrow indicates the N-wave, the black arrow indicates the P-wave. (For interpretation of the references to colour in this figure legend, the reader is referred to the Web version of this article.)

All awake patients showed a visible evoked response to the noxious electrical stimulation in the EEG (Fig. 2a–c). In Group R (Fig 2a), the N-wave increased from −9.06 μV (SD=10.63 μV) around 120 ms post-stimulus to −6.49 μV (SD=10.92 μV) at the lowest point from the awake state; this difference was not statistically significant (*P*=0.106). The magnitude of the P-wave increased slightly between conditions. The maximum value for the P-wave of 7.94 μV (SD=3.46 μV) at 297 ms in the awake state increased to a maximum value of 7.95 μV (SD=6.8 μV) at 254 ms after remifentanil administration, without significant changes.

In Group P (Fig 2b), the evoked potential completely vanished after the administration of propofol. After loss of consciousness, only alpha waves were visible in the averaged EEG. The increase at around 130 ms of the N-wave from −9.75 μV (SD=5.55) μV to −1.78 μV (SD=5.65 μV) was not statistically significant (*P*=0.156, max AUC effect size 0.083); the decrease of the P-wave at 250 ms from the maximum value of 7.55 μV (SD=3.43) μV to −0.73 μV (SD=2.85 μV) was statistically significant (*P*=0.031, max AUC effect size 0.94).

All patients (Fig 2c) lost their N- and P-waves during stable general anaesthesia with propofol and remifentanil. The disappearance of the N-wave at 130 ms, with minimum values increasing from −7.88 μV (SD=9.44 μV) to −3.58 μV (SD=5.54 μV) was statistically significant (*P*=0.002) and showed a maximum effect size of 0.20. The decrease of the P-wave around 300 ms from a maximum value of 7.34 μV (SD=3.35 μV) to −1.06 μV (SD=3.27 μV) was also statistically significant (*P*=0.001) with a maximum effect size of 0.93.

**Fig. 3:**
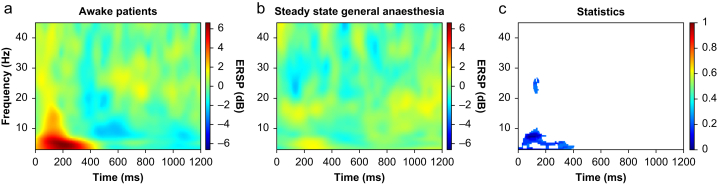
Event-related spectral perturbation (ERSP) and statistical comparison including area under the receiver operating characteristic (AUROC) curve effect sizes of the event-related data of all patients during the awake state and general anaesthesia. The panel shows the event-related spectral changes after the phase-locked noxious stimulation. The graph on the right shows the statistical comparison; a pixel is only coloured red or blue according to the colour bar if the difference is statistically significant. The colour then depicts the value of the AUROC effect size. (For interpretation of the references to colour in this figure legend, the reader is referred to the Web version of this article.)

For more detailed analysis of the time frequency domain, we also looked at the phase-locked response as spectral perturbation at the same conditions for all the patients during wakefulness and steady general anaesthesia (Fig 3), and used a statistical comparison using our AUROC model. The ERSP value of the phase-locked response between 1 and 10 Hz from approximately 0 to 400 ms, decreased significantly from 6.63 dB (SD=0.79 dB) to 5.70 dB .(SD=0.19 dB) at different time points (203 ms during wakefulness, 266 ms during steady general anaesthesia) and different points in frequency (4.46 Hz during wakefulness, 3.49 Hz during steady general anaesthesia). The minimum AUROC value in the same region was 0 [0; 0] at 7.39 Hz and 74 ms and, thus, rated as excellent on the traditional scale.

### Tetanic stimulation

**Fig. 4:**
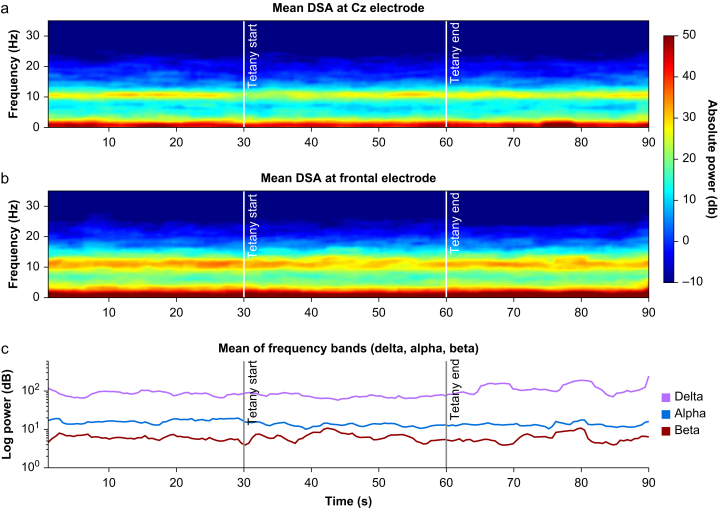
Spectral changes before, during, and after tetanic stimulation in absolute terms. The upper panel shows the changes in the absolute power in density spectral array (DSA) for electrode Cz across time averaged from seven patients, and the middle panel shows the absolute power in DSA averaged for the frontal electrodes Fp2, Fp2, and F4 from 17 patients. The black lines in all three panels indicate the start and end of the tetanic stimulation.

As ERSP and ERP vanished under clinical anaesthesia, we applied tetanic stimulation to 10 patients in Group R as a more powerful noxious stimulus and as a proxy for surgical pain.^50^ We looked at spectral changes at central and frontal electrodes and changes in the SPI as peripheral nociceptive indices. The average spectrogram across the 10 patients shows strong delta and alpha oscillations at electrode Cz that fluctuated in intensity over time (Fig 4). We visually inspected the alpha power trend (8–12 Hz) before, during, and after the tetanic stimulation in seven patients who received tetanic stimulation before and after neuromuscular block. No patient showed a visible alpha dropout^40^ (a decrease of oscillatory power). As the frontal EEG is of specific interest in the clinical setting, we present the corresponding average spectrogram for the frontal EEG in Fig 4b. The average spectrogram shows that a decrease in the absolute alpha oscillation power occurred after beginning of the tetanic stimulation at this electrode location. Changes in the EEG in response to nociceptive stimulation during general anaesthesia are described for the delta, alpha, and beta frequencies.^15^ We show the average power of these frequency ranges in Fig 4c. We did not observe any consistent changes during ongoing tetanic stimulation. At the end of the stimulus, the volatility in power of the slow delta and beta bands appeared to increase, but we refrain from drawing conclusions for our population. We did not observe any visible alpha dropouts either.^40^ We also found no signatures of burst suppression^51^ in any of the 17 individual DSA plots.

**Fig. 5:**
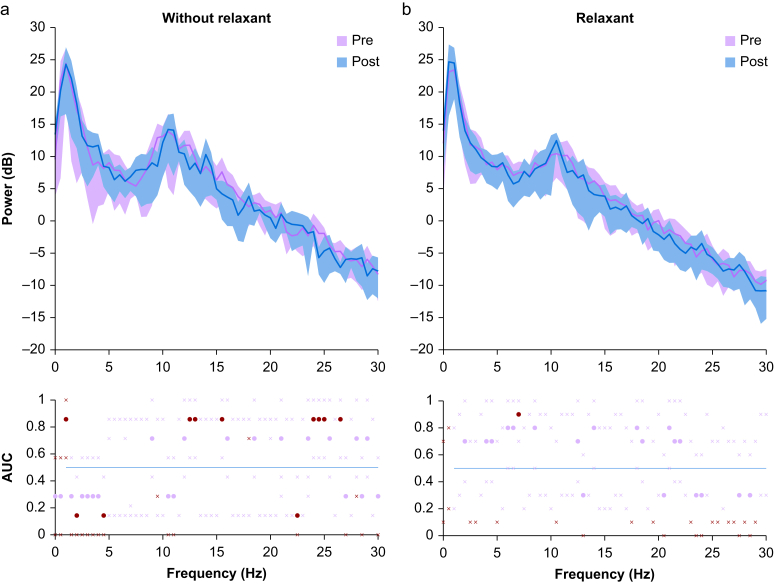
The spectral power changes either with or without neuromuscular block after tetanic stimulation. Panel (a) shows the mean power spectrum of seven patients within 10 s before (blue) and within 10 s after (grey) tetanic stimulation before neuromuscular block. Panel (b) is the same for 10 patients with complete neuromuscular block. Statistics are shown in the lower panel. AUC is calculated as individual absolute change between the prestimulation (pre) and poststimulation (post). Black dots indicate AUC effect size >0.75, grey AUC effect size <0.75. (For interpretation of the references to colour in this figure legend, the reader is referred to the Web version of this article.)

As neuromuscular block has been suggested to influence the EEG during tetanic stimuli, we considered responses without and with neuromuscular block^52^ (Fig 5a and b). We compared the power spectrum of the frontal EEG 10 s before and during the last 10 s of tetanic stimulation. Both subgroups show dominant delta and alpha oscillations. AUC analysis shows no consistent changes between pre- and poststimulation. Only the group without neuromuscular block (Fig 5a) before tetanic stimulation showed a small cluster of AUC values >0.75 in the beta region at 25 Hz which is not statistically significant. The group with neuromuscular block visually shows a lower alpha peak.

### Surgical pleth index and bispectral index

**Fig. 6:**
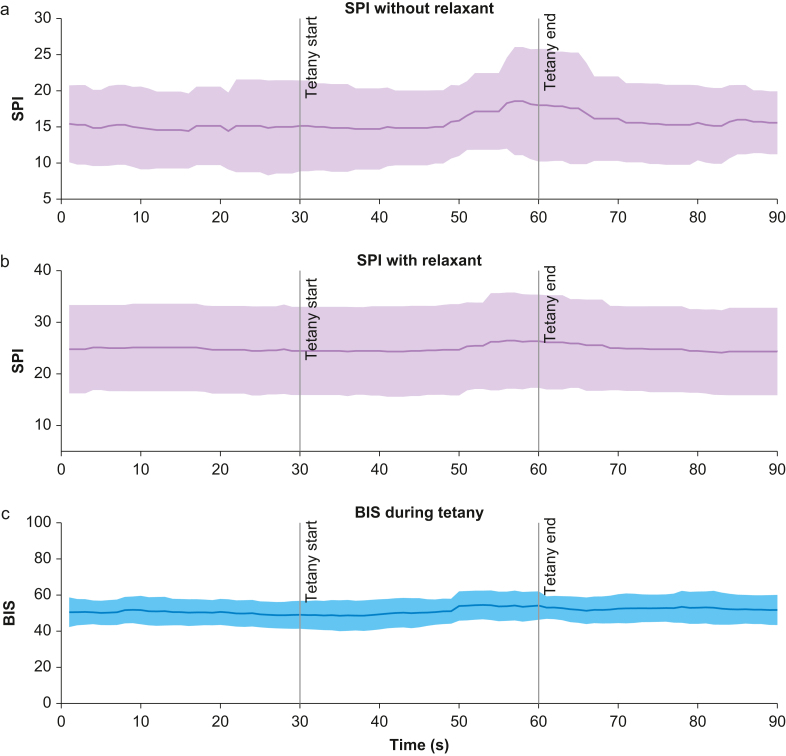
The average SPI during tetanic stimulation changes in patients without neuromuscular block. The lines indicate the average SPI or BIS values across time, while the shading indicates one standard deviation. The black bars indicate the start and end of the tetanic stimulation. As there are no absolute target values established for the SPI, normalised values are shown. Panel (a) shows SPI response of 7 stimulations before neuromuscular block, panel (b) the same of 10 stimulations at full neuromuscular block and panel (c) the average BIS of all 17 patients. BIS, bispectral index; SPI, surgical pleth index.

The average SPI value increased after ∼20 s of the onset of the tetanic stimulation without neuromuscular block (Fig 6). The mean SPI before tetanic stimulation was found to be 14 whereas the mean SPI at the end was 18 (*P*=0.035, Hedges' *g* effect size 0.32 [CI 0.12–0.77] low to medium effect). After neuromuscular block the mean SPI was 25 before tetanic stimulation and 26 at the end of the stimulation (*P*=0.11, Hedges' *g* effect size 0.11 [CI 0.01–0.42] no effect).

During tetanic stimulation, all patients had a clinically adequate and stable level of anaesthesia with a BIS value in the recommended target range for general anaesthesia (40–60), as shown in Fig 6c. During tetanic stimuli, no statistically significant difference in BIS values was observed in patients with or without neuromuscular block. Individually, we saw an increase of BIS values in two out of 17 patients during tetanic stimulation, whereas all others showed no visible change.

## Discussion

Here, we explored the integration of standardised electrical phase-locked noxious stimuli and tetanic stimulation during clinical anaesthesia. Our data show that once propofol is administered to the patient, either before or after remifentanil, the common evoked potential in the EEG after standardised noxious stimulation vanishes. Propofol, itself, has no long-lasting effect on subjective pain ratings,^53^ hence it is unlikely that the elimination of these potentials stems from an analgesic effect. It has also been shown that spinal transmission and a regional brain activity persist during intense noxious stimuli, even during deep propofol anaesthesia.^44^ Nevertheless, as there was no identifiable response in the perturbation spectrogram or the amplitude time spectrum, we conclude that it is not possible to extract any identifiable response in the EEG after repeated phase-locked noxious stimulation during routine general anaesthesia, at least with our stimulation parameters.

A recent study showed that intense noxious stimulation can lead to cortical activation, even at deep levels of anaesthesia which could cause burst suppression in the EEG.^26^^,^^44^ We could not reproduce the same somatosensory response in the EEG with our slightly lower current setting (50 mA *vs* 80 mA). Nevertheless, tetanic stimulation with 50 mA is a strong, intense noxious stimulus as shown by the response of the SPI during anaesthesia and as reported before.^50^ This was also confirmed by a significant increase of SPI in response to the tetanic stimulus in patients without neuromuscular block. In patients with neuromuscular block, this effect was not significant, raising the question of whether the effect is caused by direct nerve stimulation or the corresponding muscle contraction. Since the SPI is determined from optical pulse oximetry, we exclude a direct electrophysiological artifact. With values below 30, our SPI indicated an adequate balance of nociception-antinociception during both conditions.^24^ Normative SPI reference values for the tetanic pain model are not established and individual, relative change over time seems to be more important.^24^

Out of the many nociceptive indices, we chose the SPI because it is widely used, easy to apply, and well validated for total intravenous anaesthesia.^21^ Further detailed examination of other variables such as heart rate spectra after noxious stimulation may provide further insights.^50^^,^^54^ Other commercially available nociceptive indices measure changes in pupil width, skin conductance, or changes in reflex responses, which we are not reporting in our study. However, we believe that the choice of index used should be made with careful consideration of the patient group, the applicability, and the nociceptive stimulus used.^24^

All our patients maintained stable anaesthesia during and after tetanic stimulation, with BIS values of between 40 and 60. The BIS may be affected by beta arousal,^15^ which we observed to some extent in a non-significant fashion in the non-neuromuscular block group. This was also shown by the presence of ongoing slow delta and alpha oscillations, which predominantly serve as landmarks for deep and adequate anaesthesia,^55^ and the absence of burst suppression, which would be prominently visible in the raw EEG and spectrogram.^51^^,^^56^ Although we minimised confounding environmental influences such as noise and different times of day as far as possible, our conditions are not comparable to the laboratory conditions of a study with volunteers. We assume that pre-existing pain, as experienced by some of our patients, and the perceived stress of upcoming surgery, influence the nociceptive sensation and lead to an activation of the antinociceptive system.^57^

In our study, anaesthesia was induced with high initial doses of propofol and patients received remifentanil either immediately before or after induction, which is different to preclinical studies.^26^^,^^44^ The aim of our anaesthesia protocol was to achieve LOR and maintain anaesthesia in accordance with routine clinical practice, hence, we needed a wide range of effect-site concentrations of propofol as each patient required different doses. The order in which remifentanil and propofol were administered also played an important role as the combination of both drugs leads to synergistic effects.^58^ Although tetanic stimulation is discussed as a model for noxious incision during general anaesthesia,^50^ our data show that with our stimulation settings it is unlikely to be a suitable pain model for analysing the EEG as a nociceptive marker in clinical anaesthetic practice.

With our approach, strong tetanic stimulation may not be a general proxy of noxious stimulation as needed for studies during clinical anaesthesia before incision, but studies of larger cohorts may be worth pursuing. As the SPI depends on the reactivity of the cardiovascular system, age is likely to influence the SPI.^24^ The age range of our patients was relatively narrow. In future studies, however, age should be taken into account, especially because the influence of age on the pEEG and raw EEG has been described.^59^, ^60^, ^61^ Furthermore, no statements can be made about the impact of different types of intraoperative noxious stimulus (e.g. somatic *vs* visceral).

For future clinical studies we recommend considering the following adaptations of our protocol.

(1) Increase the number of trials for ERSP (from e.g. 5 to 100). This would ensure that the background EEG features common to general anaesthesia (dominant slow delta and alpha oscillations) would be more likely to cancel out.
(2) Increase the target effect-site concentration of propofol more slowly and, thus, achieve LOR later in the protocol. This would highlight if there were a cut-off concentration for propofol at which it eliminates the evoked potential in the EEG, as shown in studies on healthy subjects.^44^
(3) Greater consideration of neuromuscular block in electrical pain models.
(4) Increase current intensity for intense stimulation, titrated using a peripheral nociceptive index.

## Conclusions

Our data revealed that anaesthetic agents affect the cortical processing of noxious stimuli. Low doses of remifentanil alone decreased the ERSP response to noxious stimulation in our patients less than what would be expected from volunteer studies. During the alpha-dominant EEG rhythm induced by propofol, the ERSP response to noxious stimulation is masked. Strong nociceptive tetanic stimulation would more likely be detectable peripherally than in the cortical EEG of our patients. We argue that stimulation settings optimised for translation into clinical practice need to be further adapted to obtain reproducible responses to noxious stimulation in spectral EEG as found in preclinical studies. These properties are a prerequisite for a biomarker of nociception during general anaesthesia in a heterogeneous clinical patient population. We found that the peripheral nociceptive index is more sensitive to intense stimulation and could help to find standardised stimulation settings combining comparability of biomarkers and clinically relevant response. An adjustment of the stimulus intensity should be considered in future studies.

## Authors' contributions

Study design: MA, BA, MK, CW, SZ.

Patient recruitment, data collection: MA, CW, SZ.

Data analysis: MA, DH, MK, SZ.

Writing up of the first draft of the paper: MA, SZ.

Revising the manuscript critically: BA, ED, DH, MK, CW.

Interpretation of data: ED, DH, MK.

Final approval: all authors.

## Funding

The Group for Human Experimental Pain Models, Fraunhofer ITMP and the Johanna-Quandt-Jubiläumsfonds by the Johanna-Quandt-Stiftung (grant number 4.73.30). The research funding program Landes-Offensive Zur Entwicklung Wissenschaftlich ökonomischer Exzellenz (LOEWE) of the State of Hessen, Research Center for Translational Medicine and Pharmacology (TMP). The Leistungszentrum Innovative Therapeutics (TheraNova) funded by the Fraunhofer Society and the Hessian Ministry of Science and Art. BA was supported by the EKFS Research Training Group Translational Research Innovation-Pharma (TRIP).
